# Risk Factors of Severity and Mortality Among COVID-19 Patients: A Prospective Observational Study From a Tertiary Care Center

**DOI:** 10.7759/cureus.27814

**Published:** 2022-08-09

**Authors:** Bharathi Arunan, Swasthi S Kumar, Piyush Ranjan, Upendra Baitha, Gaurav Gupta, Arvind Kumar, Krithika Rangarajan, Pankaj Jorwal, Manish Soneja, Mani Kalaivani, Naveet Wig, Ashutosh Biswas

**Affiliations:** 1 Medicine, All India Institute of Medical Sciences, New Delhi, New Delhi, IND; 2 Radiology, All India Institute of Medical Sciences, New Delhi, New Delhi, IND; 3 Biostatistics, All India Institute of Medical Sciences, New Delhi, New Delhi, IND

**Keywords:** predictive model, covid-19 india, mortality rate in covid 19, observational study, risk factors of covid-19

## Abstract

Introduction

The COVID-19 pandemic has been a major public health threat for the past three years. The RNA virus has been constantly evolving, changing the manifestations and progression of the disease. Some factors which impact the progression to severe COVID-19 or mortality include comorbidities such as diabetes mellitus, hypertension, and obesity. In this study, we followed a cohort of patients to evaluate the risk factors leading to severe manifestations and mortality from COVID-19.

Methodology

We conducted a prospective observational study of 589 COVID-19 patients to assess the risk factors associated with the severity and mortality of the disease.

Results

In our cohort, 83.5% were male, with a median age (p25, p75) of 39.71 (30-48) years. The most common comorbidities included diabetes mellitus (7.8%) and hypertension (7.9%). About 41.7% had an asymptomatic disease, and of the symptomatic, 45% were mild, 6% moderate, and 7% severe. The mortality rate was 4.1%. Risk factors for severity included breathlessness (p=0.02), leukocytosis (p=0.02), and deranged renal function (p=0.04). Risk factors for mortality included older age (p=0.04), anemia (p=0.02), and leukocytosis (p=0.02).

Conclusions

COVID-19 commonly leads to asymptomatic or mild illness. The major factors we found that were associated with severity include breathlessness at presentation, leukocytosis, and deranged renal functions. The factors associated with mortality include older age, anemia, and leukocytosis.

## Introduction

The COVID-19 pandemic, which started as a cluster of influenza-like cases in Wuhan, China, continues ravaging several countries and territories. Currently, there are 520 million cases with 6.2 million deaths. It is seen that 14% of the patients develop severe disease, and the case fatality rate is around 2.2% [1]. About 3.4 million individuals have succumbed to the illness in India [2].

Some factors associated with severe COVID-19 are diabetes mellitus, smoking, obesity, malignancies, chronic kidney disease (CKD), chronic obstructive pulmonary disease (COPD), heart diseases, pregnancy, and transplants [3]. Clinical features like dyspnea and laboratory parameters like lymphopenia, thrombocytopenia, leukocytosis, elevated procalcitonin, D-dimer, aspartate aminotransferase (AST), lactate dehydrogenase (LDH), C-reactive protein (CRP), and lower levels of albumin have also been shown to be associated with increased severity and mortality [4,5]. Here, we present the data collected from our cohort of patients admitted to a tertiary care center in Northern India. We have attempted to elucidate some important clinical and laboratory parameters associated with increased severity and mortality in COVID-19 infection.

## Materials and methods

A prospective observational study was conducted at the All India Institute of Medical Sciences, New Delhi, between March and October 2020. The Institutional Ethics Committee (human research) of All India Institute of Medical Sciences, New Delhi, approved the study for research (reference number: IEC-295/17.04.2020, RP-37/2020).

Inclusion criteria

Inclusion criteria were as follows: 1. patients who tested positive for severe acute respiratory syndrome coronavirus 2 (SARS-CoV-2) by reverse transcriptase polymerase chain reaction (RT-PCR) of an upper or lower respiratory sample; 2. patients who were ≥ 18 years of age; and 3. patients who were admitted to the inpatient facility.

Exclusion criteria

Exclusion criterion was as follows: participants who denied informed consent. ​​​​​For analysis, patients were divided into non-severe (mild and moderate) and severe COVID-19 based on our institutional criteria. The definitions were as follows: mild COVID: upper respiratory tract symptoms and/or fever without shortness of breath or hypoxia; moderate COVID: evidence of lower respiratory tract illness along with respiratory rate > 24/minute and oxygen saturation (SpO_2_) < 94% on room air; and severe COVID: patients with respiratory distress requiring invasive or non-invasive mechanical ventilation or hemodynamic instability.

Data Collection

A predesigned proforma collected demographic details, comorbidities, clinical features, laboratory parameters, and outcomes. Participants were followed up daily till death or discharge.

Statistical Analysis

The data were maintained in a Microsoft Excel (Microsoft Corporation, 2019) sheet. Continuous variables were presented as median (p25, p75) or mean (standard deviation), and categorical variables were presented as numbers (percentage). Symptomatic patients were divided into the following categories: severe or non-severe (including mild and moderate disease) and survivors or non-survivors. The comparison between these groups was done using Chi-square or unpaired t-test. Odds ratio (OR) for the occurrence of severe disease and mortality was calculated. Results were considered statistically significant if p-value < 0.05. Multivariate analysis was carried out with those factors which had significant odds ratios after bivariate analysis.

## Results

Of the 589 patients included, 83.5% were male, and the median (p25, p75) age was 39.71 (30-48) years. The comorbidities in this cohort included diabetes mellitus (7.8%) and hypertension (7.9%), most commonly. Of the patients, 41.7% had an asymptomatic disease. The symptomatic individuals were classified into mild (45%), moderate (6%), and severe disease (7%). The most common presenting complaint was fever (40.9%), followed by cough and breathlessness (Table 1).

**Table 1:** Baseline characteristics of asymptomatic, mild, moderate, and severe patients COPD: chronic obstructive pulmonary disease; SpO_2_: oxygen saturation; IQR: interquartile range; PR: pulse rate; bpm: beats per minute; RR: respiratory rate.

Parameter	Asymptomatic	Mild	Moderate	Severe
Age (median (IQR))	36 (19-80)	38 (20-71)	38 (22-80)	55.5 (20-78)
Male	218 (44.3%)	219 (44.5%)	24 (4.8%)	31 (6.3%)
Female	28 (28.8%)	46 (4.42%)	12 (12.3%)	11 (11.3%)
Diabetes mellitus	6 (2.4%)	15 (5.66%)	7 (19.44%)	18 (42.8%)
Hypertension	6 (2.4%)	16 (6.04%)	6 (16.7%)	19 (45.2%)
COPD	0	1 (0.4%)	1 (2.8%)	2 (4.8%)
Chronic kidney disease	2 (0.81%)	4 (1.51%)	4 (11.11%)	7 (16.67%)
Smoking	5 (2.03%)	9 (3.40%)	2 (5.56%)	5 (11.9%)
Alcoholism	17 (6.91%)	29 (10.94%)	3 (8.33%)	5 (11.9%)
Cough	0	119 (44.9%)	24 (6.7%)	21 (5%)
Fever	0	176 (76.5%)	28 (7.8%)	26 (6.2%)
Breathlessness	0	38 (14.3%)	14 (3.8%)	31 (7.4%)
Throat pain	0	61 (23.0%)	9 (25%)	1 (2.4%)
Myalgia	0	67 (25.2%)	10 (27.78%)	3 (7.1%)
Diarrhea	0	20 (7.5%)	3 (8.33%)	1 (2.4%)
Loss of smell/taste	0	25 (9.4%)	5 (13.9%)	0
Tachycardia (PR > 100 bpm)	0	13 (4.9%)	11 (30.5%)	23 (54.7%)
Tachypnea (RR > 24)	0	0	14 (8.8%)	31 (73.8%)
Hypoxia (SpO_2_ < 94%)	0	0	17 (47.2%)	25 (59.5%)

The laboratory parameters, including complete blood counts and renal and liver function test on the day of admission, are mentioned in Table 2.

**Table 2:** Laboratory parameters of asymptomatic, mild, moderate, and severe COVID-19 patients AST: aspartate transaminase; ALT: alanine transaminase.

Parameter	Asymptomatic	Mild	Moderate	Severe
Hemoglobin (g/dL) (mean (SD))	13.6 (1.8)	13.7 (1.7)	12.2 (2.8)	10.2 (2.4)
Total leukocyte count (cells/µL) (median (p25, p75))	5,800 (1,500-61,300)	5,780 (2,510-58,000)	6,390 (1,850-13,700)	13,865 (480-59,000)
Platelet count (*10^3 ^cells/µL) (mean (SD))	2.16 (94)	2.19 (83)	2.23 (71)	1.85 (79)
Creatinine (mg/dL) (median (p25, p75))	0.79 (0.34)	0.79 (0.2-10.91)	0.8 (0.47-21.9)	1.7 (0.4-14.9)
Bilirubin (mg/dL) (median (p25, p75))	0.64 (0.21-3.52)	0.63 (0.1-6.5)	0.5 (0.21-2.32)	0.71 (0.2-18.4)
AST (U/L) (median (p25, p75))	31 (2.4-295)	36 (13.2-414)	28 (9.3-195)	57 (17-198)
ALT (U/L) (median (p25, p75))	33 (8-777)	36 (10-220)	36 (10-220)	47 (11-331)

The majority of the young and middle-aged patients had asymptomatic and mild disease, while 60% of the patients in the age group of > 60 years had severe disease. Patients with underlying comorbidities like diabetes, hypertension, COPD, AKI or CKD, and anemia had a predilection for severe COVID-19 infection. The predictors of severe disease and mortality have been depicted in Tables 3, 4.

**Table 3:** Comparison of the baseline characteristics of those with severe and non-severe disease COPD: chronic obstructive pulmonary disease; TLC: total leukocyte count; AKI: acute kidney injury; AST: aspartate transaminase; S. creatinine: serum creatinine; T. bilirubin: total bilirubin; SGOT: serum glutamic-oxaloacetic transaminase.

Parameter	Non-severe (n=301)	Severe (n=42)	p-value
Age: < 39 years	163 (54.2%)	10 (23.8%)	0.00
40-50 years	132 (43.9%)	16 (38.1%)	0.91
> 60 years	6 (1.9%)	16 (38.1%)	0.19
Smoking	11 (3.7%)	5 (11.9%)	0.61
Breathlessness	52 (17.3%)	31 (73.8%)	0.02
Diabetes mellitus	22 (7.3%)	18 (42.8%)	0.00
Hypertension	22 (7.3%)	19 (45.2%)	0.00
COPD	2 (0.6%)	2 (4.8%)	0.69
Anemia (hemoglobin < 10 g/dL)	14 (4.7%)	16 (38.1%)	0.47
Leukopenia (TLC < 4000/mm^3^)	37 (12.3%)	3 (7.1%)	0.67
Leukocytosis (TLC > 11,000/mm^3^)	9 (2.9%)	22 (52.3%)	0.02
Thrombocytopenia (platelet < 1 lakh)	37 (12.3%)	8 (19%)	0.10
AKI (S. creatinine > 1.2)	16 (5.3%)	28 (66.7%)	0.04
Hyperbilirubinemia (T. bilirubin > 1.2 mg/dL)	23 (7.6%)	7 (16.7%)	0.40
Transaminitis (SGOT > 50 IU/L)	73 (24.3%)	13 (3.1%)	0.25

**Table 4:** Comparison of baseline characteristics between survivors and non-survivors from COVID-19 TLC: total leukocyte count; AKI: acute kidney injury; AST: aspartate aminotransferase; ALT: alanine aminotransferase; S. creatinine: serum creatinine; T. bilirubin: total bilirubin.

Parameters	Survivors (n=565)	Non-survivors (n=24)	p-value
Age: < 39 years	381 (67.4%)	7 (2.9%)	0.00
40-59 years	231 (40.1%)	6 (2.5%)	0.00
> 60 years	16 (2.8%)	11 (45.9%)	0.00
Gender: Male/female	472 (83.5%)/93 (16.5%)	20 (83.3%)/4 (16.6%)	0.97
Comorbidities: Diabetes mellitus	38 (6.7%)	8 (33.3%)	0.00
Hypertension	37 (6.5%)	10 (41.7%)	0.00
Chronic kidney disease	14 (2.4%)	3 (12.5%)	0.004
Smoking	17 (3%)	4 (16.7%)	0.00
Alcoholism	50 (8.8%)	4 (16.7%)	0.19
Cough	166 (29.4%)	7 (2.9%)	0.98
Fever	226 (40%)	15 (62.5%)	0.02
Breathlessness	71 (12.6%)	15 (62.5%)	0.00
Anemia (hemoglobin < 10 g/dL)	26 (4.6%)	10 (41.7%)	0.00
Leukopenia (TLC < 4,000/mm^3^)	60 (10.6%)	2 (8.3%)	0.00
Leukocytosis (TLC > 11,000/mm^3^)	21 (3.7%)	13 (54.2%)	0.00
Thrombocytopenia (< 1,50,000)	77 (13.6%)	5 (20.8%)	0.14
AKI (S. creatinine > 1.2)	35 (6.2%)	14 (58.3%)	0.00
Hyperbilirubinemia (T. bilirubin > 1.2 mg/dL)	51 (9%)	7 (29.2%)	0.01
Transaminitis (AST > 50 IU/L/ALT > 50 IU/L)	110 (19.5%)/126 (22.3%)	7 (29.2%)/4 (16.7%)	0.01/0.67

A total of 24 patients succumbed to the illness, with a mortality rate of 4.1%. The median hospital stay duration among severe and non-severe patients was 11 days. The details of risk factors associated with severity and mortality after multivariate analysis included age > 60 years, anemia, and leukocytosis (Tables 5, 6).

**Table 5:** Factors associated with severity after multivariate analysis TLC: total leukocyte count; AKI: acute kidney injury; S. creatinine: serum creatinine.

Parameter	Non-severe (301)	Severe (42)	Odds ratio (CI)	p-value
> 60 years	6 (1.2%)	16 (38.1%)	10.6 (0.3-374.5)	0.19
Breathlessness	52 (17.3%)	31 (73.8%)	19.6 (1.7-223.1)	0.02
Leukocytosis (TLC > 11,000/mm^3^)	9 (2.9%)	22 (52.3%)	26.6 (1.6-450.8)	0.02
AKI (S. creatinine > 1.2)	16 (5.3%)	28 (66.7%)	16.5 (1.1-253.3)	0.04

**Table 6:** Factors associated with mortality from COVID-19 after multivariate analysis TLC: total leukocyte count; Hb: hemoglobin.

Parameter	Survivors (565)	Non-survivors (24)	Odds ratio (CI)	p-value
Age > 60 years	16 (2.8%)	11 (45.9%)	43.8 (1.3-1512.8)	0.04
Anemia (Hb < 10 g/dL)	26 (4.6%)	10 (41.7%)	100.7 (1.9-5326.6)	0.02
Leukocytosis (TLC > 11,000/mm^3^)	21 (3.7%)	13 (54.2%)	71.5 (1.7-2947.1)	0.02

## Discussion

In our prospective study, > 80% of the study population was male, of whom 6% had severe COVID-19 infection. No statistically significant difference in severity or mortality rates was seen among males and females. The majority of our cohort (60%) was between the ages of 20 and 40 years. Older age is one of the known predictors of severity and mortality of COVID-19, with the odds ratio in various studies ranging from 3.86 to 10.77 [6]. Of the 26 patients aged 60 years or older, 15 (57%) had a severe infection, of which 11 (42%) succumbed to the disease (OR=43.8).

In our analysis, current smokers (defined as those who have smoked > 100 cigarettes in their lifetime and continue to smoke) showed a three-time higher risk of developing severe illness, but this was not statistically significant. Similarly, other studies have shown a greater severity and mortality among smokers [6]. This may be explained by the fact that severe COVID-19 predominantly affects the lower respiratory tract. Hence, individuals with impaired alveolar epithelium or reduced ability to repair epithelium are more prone to severe disease [7]. Tobacco-related increased pulmonary angiotensin-converting enzyme 2 (ACE2) expression is also postulated to play a role [8].

About half of the persons suffering from diabetes mellitus and hypertension developed the severe disease, with 17.38% and 21.28% of the same, respectively, succumbing to the illness. In patients with elevated blood pressure, an imbalance between the renin-angiotensin-aldosterone system pathway could contribute to increased severity [9]. COPD is an independent risk factor for disease severity. Higher ACE-2 expression in COPD patients added to their reduced lung reserve has been postulated as the cause [10]. After the multivariate analysis, none of the comorbidities were found to be significantly associated with severity or mortality in COVID-19. 

Most of our patients presented with fever (67%) and cough (48%), which is typical for COVID-19 [11]. Our analysis showed that those with breathlessness at presentation had a risk of developing severe COVID-19 (OR=19.6) as demonstrated by Ioannou et al. [12]. The presence of fever > 38.5°C was more frequently observed in those with pneumonia. Studies have hypothesized that fever may indicate severe cytokine storm, with elevated interleukin 6 (IL-6) levels predicting disease severity and even mortality [11], but this was not seen in our study. The presence of high-grade fever has been reported in one previous study [13]. Another study has reported diabetes and hypertension as poor prognostic markers [14].

The median hemoglobin concentration was lower, and total leukocyte count was much higher in the severe illness group. A higher median creatinine was also noticed in individuals with moderate and severe disease. Kidney dysfunction is a known complication of COVID-19 infection, occurring in about 0.5% to 49% of patients in varying reports, and is a predictor of severity and mortality [9,12]. Similarly, liver dysfunction was also more common in the severe illness cohort. Of the various laboratory abnormalities, only leukocytosis and deranged kidney function showed a higher predisposition for developing severe COVID-19 with an odds ratio of 26.6 and 16.5, respectively. Further, anemia and leukocytosis were also found to be strongly associated with mortality, with hemoglobin of < 10 mg/dL having the highest association with mortality. The sensitive markers for severity or mortality that were shown in other studies were leukopenia or leukocytosis, thrombocytopenia, transaminitis, and acute kidney injury [15,16]. The mortality rate in our cohort of patients was 4.1%. This higher mortality rate can be explained by the fact that our center has multiple functioning ICUs and caters to a majority of moderate or severe COVID-19 patients. Our study has a limitation. The finding of the study cannot be generalized due to the varying healthcare infrastructure available in the different parts of the world.

## Conclusions

COVID-19 commonly leads to asymptomatic or mild disease. The major factors that were associated with severity include breathlessness at presentation, leukocytosis, and deranged renal functions. The factors associated with mortality include older age, anemia, and leukocytosis. Knowledge about these risk factors can help in triaging patients who are in need of admission and aiding in optimizing treatment plans. The increasing load on the health infrastructure can be reduced as well. Public health measures, like vaccination, can be targeted at the at-risk population.

## References

[1] Wu Z, McGoogan JM (2020). Characteristics of and important lessons from the coronavirus disease 2019 (COVID-19) outbreak in China: summary of a report of 72 314 cases from the Chinese Center for Disease Control and Prevention. JAMA.

[2] Jha P, Deshmukh Y, Tumbe C (2022). COVID mortality in India: national survey data and health facility deaths. Science.

[3] (2021). CDC: Cases, data, and surveillance. Control Prev.

[4] Mehta AA, Haridas N, Belgundi P, Jose WM (2021). A systematic review of clinical and laboratory parameters associated with increased severity among COVID-19 patients. Diabetes Metab Syndr.

[5] Hariyanto TI, Japar KV, Kwenandar F (2021). Inflammatory and hematologic markers as predictors of severe outcomes in COVID-19 infection: a systematic review and meta-analysis. Am J Emerg Med.

[6] Lu L, Zhong W, Bian Z (2020). A comparison of mortality-related risk factors of COVID-19, SARS, and MERS: a systematic review and meta-analysis. J Infect.

[7] Mason RJ (2020). Pathogenesis of COVID-19 from a cell biology perspective. Eur Respir J.

[8] Cai G, Bossé Y, Xiao F, Kheradmand F, Amos CI (2020). Tobacco smoking increases the lung gene expression of ACE2, the receptor of SARS-CoV-2. Am J Respir Crit Care Med.

[9] Gao YD, Ding M, Dong X (2021). Risk factors for severe and critically ill COVID-19 patients: a review. Allergy.

[10] Leung JM, Niikura M, Yang CW, Sin DD (2021). COVID-19 and COPD. Eur Sci J.

[11] Zhu J, Ji P, Pang J (2020). Clinical characteristics of 3062 COVID-19 patients: a meta-analysis. J Med Virol.

[12] Ioannou GN, Locke E, Green P (2020). Risk factors for hospitalization, mechanical ventilation, or death among 10 131 US veterans with SARS-CoV-2 infection. JAMA Netw Open.

[13] Peckham H, de Gruijter NM, Raine C (2020). Male sex identified by global COVID-19 meta-analysis as a risk factor for death and ITU admission. Nat Commun.

[14] Zhou F, Yu T, Du R (2020). Clinical course and risk factors for mortality of adult inpatients with COVID-19 in Wuhan, China: a retrospective cohort study. Lancet.

[15] Barman Roy D, Gupta V, Tomar S (2021). Epidemiology and risk factors of COVID-19-related mortality. Cureus.

[16] Bhattacharya A, Ranjan P, Kumar A (2021). Development and validation of a clinical symptom-based scoring system for diagnostic evaluation of COVID-19 patients presenting to outpatient department in a pandemic situation. Cureus.

